# Cellular Transport Mechanisms of Cytotoxic Metallodrugs: An Overview beyond Cisplatin

**DOI:** 10.3390/molecules191015584

**Published:** 2014-09-29

**Authors:** Sarah Spreckelmeyer, Chris Orvig, Angela Casini

**Affiliations:** 1 Dept. Pharmacokinetics, Toxicology and Targeting, Research Institute of Pharmacy, University of Groningen, Antonius Deusinglaan 1, Groningen 9713 AV, The Netherlands; 2 Medicinal Inorganic Chemistry Group, Department of Chemistry, University of British Columbia, 2036 Main Mall, Vancouver, BC V6T1Z1, Canada

**Keywords:** metal complexes, Pt drugs, membrane transporters, uptake, efflux, accumulation, mechanism of action, cancer, copper

## Abstract

The field of medicinal inorganic chemistry has grown consistently during the past 50 years; however, metal-containing coordination compounds represent only a minor proportion of drugs currently on the market, indicating that research in this area has not yet been thoroughly realized. Although platinum-based drugs as cancer chemotherapeutic agents have been widely studied, exact knowledge of the mechanisms governing their accumulation in cells is still lacking. However, evidence suggests active uptake and efflux mechanisms are involved; this may be involved also in other experimental metal coordination and organometallic compounds with promising antitumor activities *in vitro* and *in vivo*, such as ruthenium and gold compounds. Such knowledge would be necessary to elucidate the balance between activity and toxicity profiles of metal compounds. In this review, we present an overview of the information available on the cellular accumulation of Pt compounds from *in vitro*, *in vivo* and clinical studies, as well as a summary of reports on the possible accumulation mechanisms for different families of experimental anticancer metal complexes (e.g., Ru Au and Ir). Finally, we discuss the need for rationalization of the investigational approaches available to study metallodrug cellular transport.

## 1. Introduction

**Figure 1:**
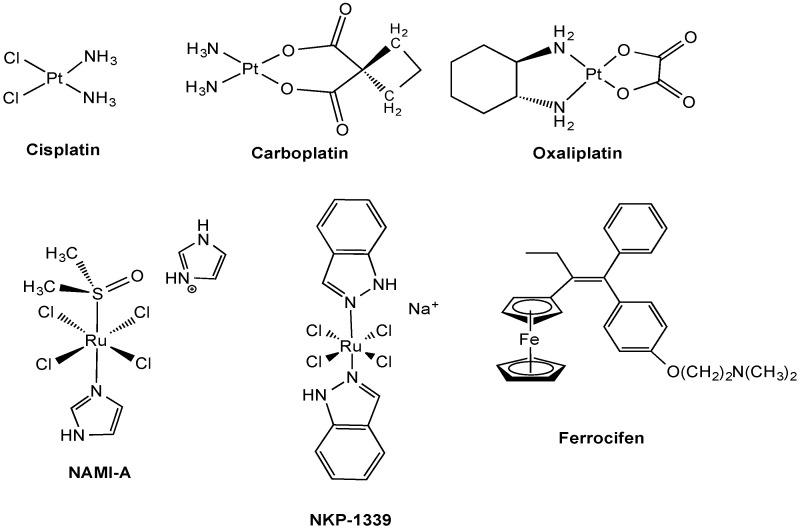
Chemical structures of clinical and experimental metal-based anticancer agents.

Cisplatin [*cis*-diamminedichloroPt(II)] (Figure 1) is an important chemotherapeutic drug used in the therapy of a broad spectrum of human malignancies such as ovarian, testicular, head and neck, and lung cancers, and in combination with a wide range of other drugs for the treatment of other malignancies. For this reason, it is one of the most widely utilized antitumor drugs in the world, with annual sales of approximately $500 million (US). Unfortunately, its use is greatly limited by severe dose-limiting side effects (nephrotoxicity, ototoxicity, and peripheral neurotoxicity) and intrinsic or acquired drug resistance. Thus, numerous Pt derivatives have been further developed with more or less success to minimize toxic effects. Over the last 30 years, 23 other Pt-based drugs have entered clinical trials with only two of these (carboplatin and oxaliplatin, Figure 1) gaining international marketing approval, and another three (nedaplatin, lobaplatin and heptaplatin) approved in individual nations [1]. Currently, there are only four Pt drugs in the various phases of clinical trial (satraplatin, picoplatin, Lipoplatin^TM^ and ProLindac^TM^).

Over the years, research on innovative anticancer metallodrugs has produced several ruthenium-based compounds as alternatives to Pt compounds. The Ru(III) complex *trans*-[tetrachloro(DMSO)(imidazole)ruthenate(III)] (NAMI-A) (Figure 1) was demonstrated to have high selectivity for solid tumor metastases and low toxicity at pharmacologically active doses [2]. This is the first ruthenium complex to enter clinical trials. A related Ru(III) compound, indazolium *trans*-[tetrachlorobis(1*H*-indazole)ruthenate(III)] (KP1019) and its sodium salt analogue NKP-1339 (Figure 1), also entered clinical trials after they were found to exhibit cytotoxic activity *in vitro* in cisplatin-resistant human colon carcinoma cell lines and *in vivo* in various tumor types [3]. Due to its higher water solubility, NKP-1339 has now been selected as a lead candidate for further clinical development.

Besides these coordination compounds, several classes of other metal complexes and organometallic compounds, based on different metals such as Au, Fe, Ag, Ga, Rh, and Ti, exhibit promising anticancer activity at least in preclinical studies [4,5,6,7,8]. Notably, Jaouen and co-workers have developed organometallic ferrocene-modified tamoxifens (named ferrocifens, Figure 1) as estrogen-targeting molecules effective in hormone-independent breast cancer cells, where hydroxytamoxifen and ferrocene are inactive [9]. In this compound class the β-phenyl ring of tamoxifen has been substituted by a ferrocenyl moiety using classical organic/organometallic synthetic methods. Such structural modifications lead to more lipophilic compounds able to easily cross cell membranes, and, therefore, provide stronger cytotoxic effects.

For most of these cytotoxic, metal-based compounds, the mechanisms leading to their pharmacological and toxicological profiles are still not fully elucidated and different biological targets have been proposed, most of which still need validation. Due to the fact that DNA was identified early as the primary target of Pt(II) anticancer agents—adduct formation causes changes in DNA structure, hindering replication and transcription, which ultimately results in the induction of apoptosis—nucleic acids and their “alkylation” were believed to constitute the main pathway of activity for any cytotoxic metal compound [10].

Therefore, until 2006, only a limited number of biophysical studies encompassed the interactions of anticancer metallodrugs with proteins. These studies mostly concerned the two major serum proteins, albumin and transferrin, involved in the transport of metals and metallodrugs in the bloodstream, as well as metallothioneins, small, cysteine-rich intracellular proteins, primarily involved in storage and detoxification of soft metal ions. Afterwards, the crucial role of the interactions of metallodrugs with protein targets in determining the compounds’ pharmacological action, uptake and biodistribution, as well as their overall toxicity profile, was fully recognized and, as a result, the number of studies increased exponentially [11,12,13]. Nowadays, cisplatin and other metal-based compounds are known to bind to several classes of proteins with different roles, including transporters, antioxidants, electron transfer proteins, and DNA-repair proteins, as well as proteins/peptides simply used as model systems to characterize the reactivity of metallodrugs *in vitro*, but that are also present *in vivo* [13].

Among the various families of investigated proteins, we believe that membrane transporters are of particular relevance. In fact, as essential mediators of specific cellular uptake, they are implicated not only in the pharmacological effects, but also in determining side-effects, metabolism, and excretion of many drugs, including cisplatin and other cytotoxic metal compounds [14]. Recent progress has been made in understanding the role of membrane transporters in drug safety and efficacy. In particular, more than 400 membrane transporters organized in two major superfamilies—ATP-binding cassette (ABC) and solute carrier (SLC)—have been annotated in the human genome [15,16]. Many of these transporters have been cloned, characterized and localized to tissues and cellular membrane domains in the human body. In drug development, particular attention has been paid to transporters expressed in the epithelia of the intestine, liver and kidney, and in the endothelium of the blood–brain–barrier (BBB). As a result, a number of studies focus on the interaction of drugs and their metabolites with mammalian transporters present in epithelial and endothelial barriers. Interestingly, clinical pharmacokinetic drug-drug interaction (DDI) studies have suggested that transporters often work together with drug-metabolizing enzymes (DMEs) in drug absorption and elimination.

In spite of their great importance, transport mechanisms of anticancer metallodrugs have not yet been fully elucidated, especially in the case of a new generation of cytotoxic metal complexes. Moreover, the lack of a systematic investigation/approach to study metal compound accumulation in cells/tissues, as well as the generally limited structural information on membrane transporters, prevent the understanding of the complex mechanisms of drug absorption and excretion, particularly in medicinal inorganic chemistry. In this review we present a summary of the literature on the mechanisms of cellular uptake and accumulation for cisplatin, and analogues in the clinic. Most importantly, we will attempt an overview of the studies available for other families of experimental anticancer metal compounds, focusing on ruthenium and gold complexes (both coordination and organometallics) for which some studies are available in the literature. We have also considered the case of iridium-based organometallic complexes since recent detailed studies on their possible transport mechanisms have appeared. In the last section, the use of bioactive ligands to enhance the cellular uptake of metal compounds will be presented.

## 2. Transport Processes of Metal-Based Compounds

Initially, passive diffusion through the cellular lipid bilayer was considered to be the dominant process involved in drug uptake and distribution; however, recently the concept of carrier-mediated active uptake of commonly prescribed drugs has become the rule rather than the exception [14]. Thus, membrane transporters and channels, collectively termed the *transportome* [17], are increasingly recognized as important determinants of tumour cell chemosensitivity and chemoresistance. For example, reduction in Pt concentration of 20%–70% has been observed in cancer cell lines resistant to cisplatin. This reduced accumulation can result from decreased influx or increased efflux, or both. Herein, we overview studies of cellular accumulation of Pt compounds, as well as of new anticancer metal complexes; thus, the few available studies reporting on the possible accumulation mechanisms for different families of experimental anticancer metal complexes (e.g., Ru, Au and Ir) will be summarized. Most importantly, we will attempt a rationalization of the investigational approaches available to study metallodrugs cellular transport and will comment on the relation between compound accumulation and anticancer properties.

### 2.1. Anticancer Pt Drugs

Experimental evidence, well reviewed by Hall *et al*. [18] has led to the conclusion that cisplatin most likely enters the cell via two pathways: (a) passive diffusion and (b) facilitated and active uptake by a number of transport proteins [19]. Membrane transporters of Pt-based anticancer agents determining active Pt uptake and efflux pathways, as well as their clinical significance have also recently been reviewed by Burger *et al*. [20] including Cu transporters (Ctrs) organic cation transporters (OCTs), solute carriers (SLCs) and ATP-binding cassette (ABC) multidrug transporters. Other studies pointed also towards the involvement of different transport mechanisms in the overall biological activities of platinum compounds, including Na^+^-dependent glucose transport [21], and other ATP-dependent processes beside those regulated by Na^+^,K^+^-ATPase [22]; however, no actual validation of such mechanisms have been attained so far. Our review presents a selection of *in vitro*, *in vivo* and clinical studies that elucidate mechanisms of accumulation for cisplatin and related Pt(II) anticancer drugs, together with information on the structural features and tissue distribution of the mentioned uptake and efflux transporters. When available, information of the reactivity of metal compounds with the protein transporters at a molecular level obtained by biochemical and biophysical methods will be provided.

#### 2.1.1. Cu Transporters

**Figure 2:**
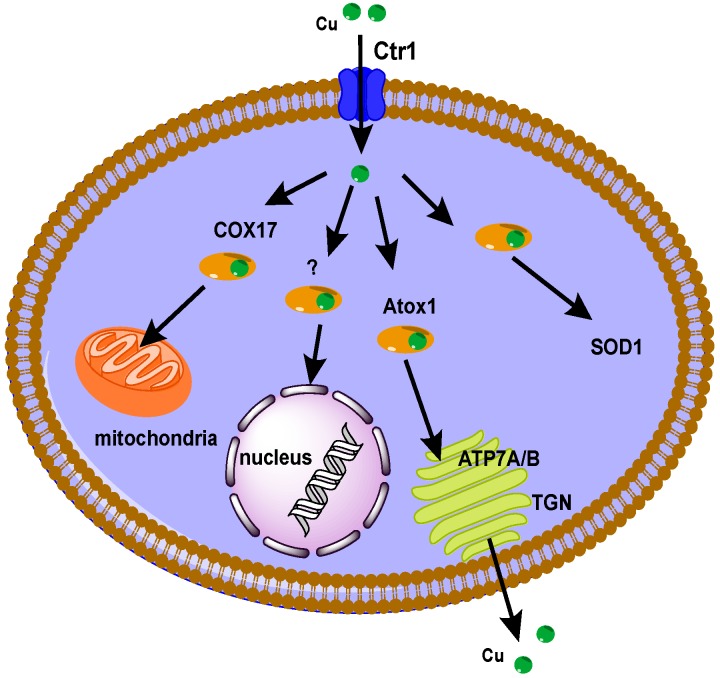
Schematic drawing of the major intracellular human Cu trafficking pathways.

According to the Human Genome Organisation (HUGO), human transporters are classified based on their amino acid sequence in 43 solute carrier (SLC) families [23]; Cu transporters have been assigned to the SLC31A family. Cu is an essential nutrient for almost all eukaryotic organisms to effect biological processes (e.g., free radical detoxification, mitochondrial respiration, iron metabolism, biosynthesis of neuroendocrine peptides *etc*.) and is a cofactor in many enzymes. Due to the fact that the intracellular form of Cu, Cu^+^, is highly toxic because it reacts with molecular oxygen or hydrogen peroxide to produce free radicals, Cu homeostasis is guaranteed by a complex network of proteins that bind and deliver Cu^+^ to the Cu-dependent proteins and protect cells from the harmful effects of excess “free” Cu. Figure 2 is a schematic diagram of the Cu homeostasis system in mammals. Cu^+^ enters cells via the 23 KDa channel-like Cu transporter 1 (Ctr1) and is handed to pathway-specific chaperones such as antioxidant protein 1 (Atox1), Cu chaperone for superoxide dismutase (CCS), and cytochrome c oxidase assembly homolog (COX-17) that delivers it to various organelles for transfer to Cu-requiring enzymes. Afterwards, the P1B type ATPases ATP7A and ATP7B positioned in the trans-Golgi network secrete Cu. It is worth mentioning that the discovery of an existing protein homologue of Ctr1, namely Ctr2, presents another potential player in Cu homeostasis [24]. Up to now the physiology and mechanism of Cu transport via Ctr2 has been poorly understood. In mammalian cells Ctr2 localizes to intracellular vesicular compartments including endosomes and lysosomes; however, when overexpressed via transfection with an epitope tag attached to the protein, Ctr2 has been localized at the plasma membrane, similarly to Ctr1.

Over several years, Cu transporters have been proposed to be involved in cellular import and export of Pt(II) chemotherapeutic agents, as well as in their resistance mechanisms [19,25]. In particular, expression of the human Cu transporter 1 (hCtr1) is thought to result in increased sensitivity to cisplatin, whereas expression of two Cu(I) proteins exporting ATPase, *i.e*., ATP7A and ATP7B, is believed to be involved in the resistance to cisplatin, either by sequestering drug away from its targets (ATP7A), or by exporting the drug from the cell (ATP7B).

The human hCtr1 (SLC31A1) is an evolutionarily conserved Cu influx transporter present in plants, yeast, and mammals, and the main Cu importer in mammalian cells. It is also a key player in the homeostatic regulation of intracellular Cu levels to ensure that nutritional delivery of Cu to enzymes, such as cytosolic Cu,Zn-superoxide dismutase (SOD). hCtr1 is located in the plasma membrane and is constituted by three transmembrane helices, an extracellular N-terminal domain and a cytosolic C-terminal domain [26]. Three hCtr1 molecules form a channel-like symmetric trimer, as revealed by electron microscopy. hCtr1 contains two Met-rich motifs and two His-rich motifs on its extracellular N-terminus; both are thought to be essential for the function of the transporter [27]. Interestingly, the Met-rich motifs located in the N-terminal domain and in the inner side of the channel pore are critical for the binding of Cu [28].

Studies (more than a decade old) revealed Ctr1 to be a significant pathway for the import of Pt cancer therapeutics into both yeast and mammalian cells. Thus, hCtr1 has been proven to play an essential role in the cytotoxic effects of Pt(II) drugs in cancer cells. For example, enhanced expression of the human Ctr1 gene in cancer cells normally resistant to cisplatin (including small cell lung cancers SCLC, and SR2 cells) led to an accumulation of cisplatin, carboplatin, and oxaliplatin, suggesting that hCtr1 can transport not only cisplatin but also carboplatin and oxaliplatin, albeit at reduced rates [27]. In the same study, it was also shown that, although oxaliplatin transport was increased in cisplatin-resistant cells, the enhanced oxaliplatin accumulation failed to sensitize SR2 cisplatin-resistant cell lines, demonstrating that other intracellular pathways contribute to resistance mechanisms.

Always at a cellular level, studies of the pharmacology of cisplatin in human ovarian carcinoma A2780 cells, molecularly engineered to express increased hCtr1, showed that the overexpression of the transporter led to an increase in Pt accumulation and a decreased cell growth rate, yet it had limited effect on the sensitivity to cisplatin and to the amount of Pt binding to DNA [29]. This discrepancy suggests that much of the Cu and cisplatin conducted into the cell must be sequestered away from the target through which it triggers cytotoxicity. Similarly, Beretta *et al*. reported that, whereas transfecting expression human hCtr1 cDNA into cisplatin-resistant epidermoid carcinoma A431 cells conferred increased uptake of Cu, no changes in cisplatin uptake and cellular sensitivity to the drug were observed [30]. In parallel, the absence of the transporter renders cells resistant to cisplatin and carboplatin as demonstrated by Holzer *et al*., in genetically engineered murine embryonic fibroblasts (Ctr1-/-) [31]. Moreover, these last results provide strong evidence that, at concentrations <1 μM, Ctr1 mediates cellular accumulation of all three clinically used Pt-containing drugs currently used in patients, but oxaliplatin differs from cisplatin and carboplatin in that its dependence on Ctr1 diminishes at higher concentrations. Overall, the idea that these drugs may have different influx transporters is consistent with their different spectra of action against various types of human cancer.

It must be mentioned that the interplay between Pt drugs, hCtr1 and Cu is more complicated than expected. As an example, another study by Holzer, Howell *et al*., showed that cisplatin rapidly triggers loss of endogenously and exogenously expressed hCtr1 in both human ovarian cancer cell lines A2780 and 2008, and that this effect has functional consequences for the uptake of Cu [32]. Moreover, the same authors provided evidence that cisplatin-induced loss of hCtr1 in 2008 cells involves internalization from the plasma membrane by macropinocytosis followed by proteasomal degradation [33].

An original study by Chen *et al*. [34] using transfected cells displaying elevated levels of GSH, showed that such cells exhibited marked sensitivity to cisplatin due to up-regulation of hCtr1. Following these intriguing results, in 2012 Liang, Kuo *et al*., demonstrated in multiple cell models that expression of hCtr1 in cisplatin resistant variants can be preferentially up-regulated by Cu-lowering agents (chelators) as compared with those in their drug-sensitive counterparts, providing greater sensitivity to the killing by Pt drugs [35]. The parallel with the previous study by Chen [34] lies in the fact that GSH is also an abundant physiologic Cu chelator. In that same study, enhanced cisplatin efficacy by a Cu-lowering agent was also observed in animal tumor xenografts bearing cisplatin resistant cells. Furthermore, analysis of a public dataset concluded [35] that ovarian cancer patients with elevated expression levels of hCtr1 in their tumors had more favourable treatment outcomes after Pt-drug treatment than did those with low hCtr1 levels. Together, these findings provide a mechanistic basis for overcoming cisplatin resistance using Cu chelation strategy. Interestingly, the same authors conducted a pilot clinical study using the Cu-lowering agent trientine in combination with carboplatin in five Pt-resistant high-grade epithelial ovarian cancer patients [36]. Encouraging results were obtained showing preliminary clinical evidence that the role of decreasing Cu levels in reversing Pt resistance merits additional clinical investigation.

Interestingly, patients with high levels of Ctr1 in their tumor appear to respond better to drug treatment [35]. Notably, 22 single nucleotide polymorphisms (SNP) of Ctr1 have been identified by the screening of 282 non-small-cell lung carcinoma (NSCLC) Chinese patients [37]. In particular, genetic polymorphisms of Ctr1 at rs7851395 and rs12686377 were associated with Pt resistance in NSCLC patients. Thus, these findings corroborate the idea that Ctr1 plays an essential role in Pt resistance and that it could be considered a predictive marker for the pretreatment evaluation of NSCLC patients.

Very recently, Kim *et al*. [38] compared tumor Ctr1 expression with intratumoral Pt concentration in clinical specimens. In detail, these authors have hypothesized that a defect in tumor Ctr1 expression is associated with reduced tissue Pt accumulation and tumor response in NSCLC following Pt-based chemotherapy. The study showed that NSCLC patients with undetectable Ctr1 expression in their tumors had reduced intratumoral Pt concentration and tumor response compared to patients with any level of Ctr1 expression. Unfortunately, this study enrolled a limited number of patients and independent validation with a prospective clinical trial would be necessary [38].

Concerning side-effects of Pt drugs, renal tubular damage is recognized as a major pathogenic factor in cisplatin nephrotoxicity. In fact, cisplatin is accumulated in renal tubular cells at high concentrations, leading to tubular injury and cell death. Recent research has revealed multiple signalling pathways that are responsible for tubular cell injury and death during cisplatin nephrotoxicity. In 2009 Pabla *et al*., demonstrated that Ctr1 is mainly expressed in both proximal and distal tubular cells in mouse kidneys [39]. Importantly, down-regulation of Ctr1 in human embryonic kidney (HEK293) by small interfering RNA or Cu pre-treatment resulted in decreased cisplatin uptake [39]; however, cimetidine, a substrate of organic cation transporters (OCTs), also had partial inhibitory effects on cisplatin uptake in HEK293 cells. Notably, it was also shown that cimetidine could further reduce cisplatin uptake in Ctr1 knockdown HEK293 cells, and both apoptosis and necrosis induced by cisplatin were further reduced by cimetidine in Ctr1 knockdown HEK293 cells [39]. According to these results, it could be hypothesized that not only Ctr1 but also a cimetidine-inhibitable transport system, probably an organic cation transporter, contribute to cisplatin transport in renal tubular cells, resulting in nephrotoxicity. Nevertheless, it must be considered that cimetidine may also have off-target effects.

Interestingly, cisplatin treatment of a cell line expressing hCtr1 revealed the time- and concentration-dependent appearance of a stable hCtr1 multimeric complex, consistent with a homotrimer, that was not observed following Cu treatment of these same cells [40]. Mutagenesis studies identified two methionine-rich clusters in the extracellular amino-terminal region of hCtr1 that were required for stabilization of the hCtr1 multimer by cisplatin, suggesting that these sequences bind cisplatin, and, subsequently, form crosslinks between hCtr1 polypeptides [40].

At a molecular level, Natile and co-workers investigated the binding of Pt complexes to the Met-rich domain of hCtr1 by different techniques including mass spectrometry and NMR spectroscopy [41]; according to their findings cisplatin appears to easily form adducts with the peptide domain in which all the original ligands of Pt are lost and replaced by the S-donor Met groups. Based on these observations, cisplatin would be actually sequestered by hCtr1 and not transported, while a possible transport system could actually be an endocytotic process, incorporating a portion of the extracellular milieu (containing non-degraded cisplatin) into vesicles, which are subsequently delivered to subcellular compartments. These latter results might be in accordance with a more recent paper reporting on the fact that overexpression of hCtr1 in the human embryonic kidney HEK293 cell line did not result in increased sensitivity to cisplatin [42]. Other studies based on MS and NMR spectroscopy characterized the binding of Pt compounds with synthetic peptides corresponding to hCtr1 Met-motifs at a molecular level, in some cases highlighting the differences among the various Pt drugs [43,44]. Finally, recent studies by Wang *et al*., based on NMR spectroscopy and electrospray ionization mass spectrometry (ESI-MS) show that a maximum of two Pt atoms is bound to each monomer unit of hCtr1 for cisplatin, carboplatin and nedaplatin [45].

Although many different systems appear implicated in cisplatin trafficking, once Pt enters the cells, evidence suggests a linkage between cisplatin resistance and the human Cu homeostatic proteins Atox1 and ATP7A or ATP7B [46]. The Cu chaperone Atox1 binds Cu(I) at a conserved CXXC motif and delivers it to the N-terminal metal binding domains (MBDs) of ATP7B and ATP7A, which are Cu(I) specific P1B-type ATPases. Each human Cu(I) ATPase has six MBDs, which also bind Cu(I) with CXXC motifs and resemble Atox1 in the overall structure [47]. The structure of a stoichiometric cisplatin-Atox1 adduct (Pt-Atox1) was determined at 1.6 Å resolution showing a Pt(II) ion coordinated to Cys12 and Cys15 from the CXXC motif [48]. The geometry is square planar with the two cysteine ligands oriented *trans* to one another. The remaining ligands are provided by the backbone amide nitrogen of Cys12 and a 2-carboxyethylphosphane (TCEP) molecule with a TCEP(P)-Pt distance of 2.48 Å. In the same paper the structure of a dimeric cisplatin adduct Pt-(Atox1)_2_ was also reported at 2.14 Å resolution. Overall, the two structures support the idea that the cisplatin interaction with Cu(I) binding motifs may lead to unfavourable therapeutic outcomes, not only due to unproductive cisplatin trafficking, but perhaps also as a result of aberrant Cu(I) transport in cisplatin resistant tumours.

Several studies support the hypothesis that both ATP7A and ATP7B are involved in the resistance to cisplatin, either by sequestering drug away from its targets (ATP7A), or by exporting the drug from the cell (ATP7B). In eukaryotes, Cu(I) ATPases both efflux excess Cu and shuttle Cu to the secretory pathway for incorporation into enzymes [47]. Mutations in the human Cu(I) P1B ATPases, ATP7A and ATP7B, lead to the Cu metabolic disorders Menkes syndrome and Wilson disease, respectively [49]. The Cu(I) ATPases consist of eight transmembrane helices, an ATP binding domain that comprises a nucleotide binding domain (N domain) and a phosphorylation domain (P domain) containing an invariant DKTGT sequence that becomes phosphorylated at the aspartate residue during the ATP hydrolysis cycle. An A-domain, a key link in coupling nucleotide hydrolysis to ion transport, is also present. ATP7A is expressed in many tissues, while ATP7B is mainly present in liver and brain. The concept that Cu exporters may mediate cisplatin resistance was introduced by Komatsu *et al*. [50], reporting cisplatin resistance in prostate carcinoma cells overexpressing ATP7B. Afterwards, several other studies, in cancer cells resistant to cisplatin, demonstrated that these cell lines overexpress at least one of the two efflux Cu transporters [51,52,53,54]. Interestingly, ATP7B siRNA incorporated into the neutral nanoliposome 1,2-dioleoyl-*sn*-glycero-3-phosphatidylcholine was highly effective in reducing tumor growth in combination with cisplatin in two orthotopic mouse models of ovarian cancer (70%–88% reduction in both models compared with controls) [52]. This reduction in tumor growth was accompanied by reduced proliferation, increased tumor cell apoptosis, and reduced angiogenesis [52].

*In vivo* studies of ATP7B expression in nine human NSCLC xenografts using real-time polymerase chain reaction (PCR) and immunohistochemistry, showed that ATP7B mRNA expression was significantly correlated with cisplatin sensitivity [55]. Moreover, ATP7B mRNA and protein expression levels in the cisplatin-resistant xenografts were significantly higher than those in the cisplatin-sensitive xenografts [55]. These results suggest that ATP7B is a cisplatin-resistance marker in human NSCLC xenografts *in vivo*. Notably, a clinical study showed that ATP7B mRNA and protein expression in colorectal tumors is associated with clinical outcomes to oxaliplatin combination therapy with 5-fluorouracil [56].

Using genomic analysis, it was found that ATP11B gene expression was substantially increased in cisplatin resistant cells [57]. Moreover, ATP11B enhanced cisplatin efflux and ATP11B silencing restored sensitivity of ovarian cancer cells to cisplatin. ATP11B is included in the sub-family 4 of P-type ATPase. These proteins are thought to translocate phospholipids, rather than cations, from the outer to the inner leaflet of membrane bilayers. One hypothesis is that this type of ATPase may be involved in the vesicular transport of cisplatin.

Finally, at a molecular level, it has been demonstrated that, similar to Cu, Pt binds the CXXC motives of the cytosolic N-terminal binding domain of ATP7B [58], and that such interaction mediates cancer cell resistance to cisplatin. Recently, it has been shown by solid supported membrane technique (SSM) that Pt drugs can activate Cu-ATPases in microsome samples and undergo ATP-dependent translocation in a fashion similar to Cu [59].

#### 2.1.2. Organic Cation Transporters (OCTs) and Multidrug and Toxin Extrusion Proteins (MATEs)

The organic cation transporters have been assigned to the solute carrier SLC22A family consisting of three sub-categories based on the charge of the transporter: the electrogenic transporter (OCT1-3), electroneutral organic cation/carnitine transporter (OCTN1-3) and the organic anion transporter (OATs, and urate transporters, URAT-1) [15]. Each transporter of the SLC22A family consists of 12 α-helical transmembrane domains (TMDs), a large glycosylated extracellular loop between TMDs 1 and 2, and a large intracellular loop between TMDs 6 and 7 with consensus sequences for phosphorylation. Most endogenous or exogenous substrates for OCTs are charged positively at physiological pH 7.4, and the electrochemical gradient is the crucial force for the uptake of the cationic substrates. The transport of organic cations is also independent of Na^+^ and reversible with respect to direction. A disadvantage of OCTs is that they are polyspecific, meaning that through their large binding domain, which contains partially overlapping interaction domains, a wide catalogue of substrates can be transported.

Many transporters of the SLC22A family are located in secretory organs such as liver and kidney, as well as intestine; therefore, they play a pivotal role in drug adsorption and excretion, and different OCTs show species- and tissue-specific distributions. For example, the human OCT1 is highly expressed in the sinusoidal membrane of the liver and in jejunum. Instead, human OCT2 is mainly expressed in the basolaterial side of renal proximal tubule cells, and in the dopaminergic brain regions. For the correct interpretation of translational studies, it is important to mention that in rodents, both OCT1 and OCT2 show a high renal expression in the basolateral membrane of proximal tubule cells, with higher OCT2 expression in male animals. hOCT3 shows a much broader tissue distribution, including skeletal muscle, heart, brain, and placenta, but the distribution in the membrane and physiological role of OCT3 are not yet clearly understood.

Investigating the mechanisms of cisplatin nephrotoxicity has evinced the role of organic cation transporters (OCTs) in cisplatin transport. Various studies demonstrating that cisplatin can be transported by OCTs in cells were based on competition experiments with other established OCTs substrates such as tetraethylammonium (TEA) and cimetidine among others. Ciarimboli and coworkers have recently extensively reviewed these studies and the reader is referred to their review papers for details [60,61]. As a representative example, interaction of cisplatin with hOCT2 in kidney or hOCT1 in liver was investigated with the fluorescent cation 4-[4-(dimethyl-amino)styril]-methylpyridinium (ASP) in stably transfected HEK293 cells and for the first time in tissues physiologically expressing these transporters, human proximal tubules, and human hepatocyte couplets [62]. Cisplatin inhibited ASP transport in hOCT2-HEK293 but not in hOCT1-HEK293. In human proximal tubules the drug competed with basolateral organic cation transport, whereas it had no effect in tubules from a diabetic kidney or in hepatocytes. In hOCT2-HEK293 cells, 15 h incubation with cisplatin induced apoptosis, which was completely suppressed by simultaneous incubation with the hOCT2 substrate cimetidine [62]. These findings support the idea of the interaction of cisplatin with hOCT2 in renal proximal tubules, but not with hOCT1, explaining its organ-specific toxicity.

More recently, the functional effects of cisplatin treatment on kidney (24 h excretion of glucose, water, and protein) and hearing (auditory brainstem response) were studied in wild-type and OCT1/2 double-knockout mice [63]. No sign of ototoxicity and only mild nephrotoxicity were observed after cisplatin treatment of knockout mice. Co-medication of wild-type mice with cisplatin and the organic cation cimetidine resulted in protection against ototoxicity and partly against nephrotoxicity [63]. Moreover, it should be noted that in rats, treatment with both cisplatin and cimetidine did not interfere with the antitumoral activity of the Pt drug [64]. Based on these studies, among others, hOCT2 has been proposed as a target for protective therapeutic interventions accompanying cisplatin treatment.

Concerning other anticancer Pt drugs, Yonezawa *et al*., demonstrated that oxaliplatin’s toxicity and uptake are also enhanced by hOCT2 expression and weakly by hOCT3 in HEK293 cells [65], while no effect was observed in the case of carboplatin and nedaplatin. Oxaliplatin, however, showed almost no influence on the TEA uptakes in the HEK293 cells expressing hOCT1, hOCT2, and hOCT3 [65]. Recently, hOCT3 appeared to be also involved in the transport of cisplatin because cisplatin-sensitive cervical adenocarcinoma KB-3-1 cells express much higher level of hOCT3 than their Pt(II) resistant variants [66]; however, studies on hOCT3-overexpressing HEK293 cells showed no effect on cisplatin accumulation with respect to wild-type cells [65]. Therefore, these results point to differences in Pt accumulation due to the selected cell type.

Concerning hOCT3, selective induction of hOCT3 mRNA expression in colon cancer and colorectal cancer-derived cell lines has been reported [67]. Interestingly, in this study the cytotoxicity and accumulation of Pt caused by the treatment with oxaliplatin, but not cisplatin, depended on the expression of hOCT3 mRNA. Thus, the uptake of oxaliplatin into the cancer cells via hOCT3 was suggested to be an important mechanism for its cytotoxicity, and the expression of hOCT3 in cancers was proposed to become a marker for including oxaliplatin in cancer chemotherapy.

Beside the OCTs, also the multidrug and toxin extrusion proteins (MATEs) are part of organic cation homeostasis, and belong to the SLC47 family. Specifically, MATEs act as H^+^/organic cation antiporters, transporting protons from the extracellular side to the cytoplasm while organic cations are exported. Two isoforms are known, SLC47A1 (MATE1) and SLC47A2 (MATE2-K). MATE1 is primarily expressed in the liver and kidney, while MATE2-K exhibits a kidney-specific expression [68]. In the liver, MATE1 is localized on the canalicular membrane of hepatocytes and appears to form a functional unit with the basolaterally expressed OCT1 to mediate the biliary excretion of cationic drugs and their metabolites across the hepatocytes. In the kidney, MATE1 is highly expressed on the luminal membrane of the proximal tubular cells and is thought to play a key role in the excretion of organic cations. In the proximal tubule epithelium MATE1 cooperates with the basolaterally expressed OCT2 in the renal secretion of organic cations. Substrates for MATE1 and MATE2-K are typical organic cations, TEA, 1-methyl-4-phenylpyridinium (MPP), metformin, cimetidine, procainamide among others. Some compounds were reported to be specific inhibitors of MATE, although OCT and MATE are common in substrate specificity.

The few reports dealing with Pt drug accumulation by MATEs, have been well summarized by Ciarimboli [69]. As a representative example of the knowledge in this area we decided to discuss the previously mentioned paper by Yonezawa *et al*. [65]. In this study, when HEK293 cells, transiently expressing hMATE1 or hMATE2-K, were treated with 50 to 1000 μM cisplatin for 2 h, the expression of the transporters did not affect cisplatin-induced cytotoxicity. In addition, the transporter activities were confirmed by the uptake of [^14^C]TEA; nevertheless, the accumulation of cisplatin was enhanced by hMATE1 more than hMATE2-K. Conversely, the accumulation of oxaliplatin was enhanced by hMATE2-K more than hMATE1 [65]. Since oxaliplatin is only poorly nephrotoxic, the obtained results suggest that the basolateral hOCT2 is the influx transporter responsible of oxaliplatin-induced toxicity, while the apical hMATE1 and hMATE2-K are efflux transporters as a means to protect cells. Overall, transcellular transport and cellular toxicity of oxaliplatin should be further examined to validate such a hypothesis.

Yokoo *et al*. [70] reported *in vivo* studies in rats where higher accumulation of cisplatin in rat kidney tissues was observed in comparison to that of either oxaliplatin or carboplatin. As expected, such higher accumulation of cisplatin led also to an increase in nephrotoxicity, which was proven by overexpression of biomarkers of kidney injury like osteopontin in kidney slices. Moreover, *in vitro* studies showed that rat MATE1 as well as human MATE1 and MATE2-K, stimulated the H^+^-gradient-dependent antiport of oxaliplatin, but not of cisplatin, carboplatin and nedaplatin [70].

Finally, a recent study by Li *et al*. [71] reported the effects of coadministering cisplatin with the antiemetic 5-hydroxytryptamine-2 (5-HT3) receptor antagonist ondansetron. The introduction of 5-HT-3 receptor antagonists has been a significant clinical advance in the prevention and treatment of chemotherapy-induced nausea and vomiting, particularly for patients receiving highly emetogenic cisplatin-based regimens. Interestingly, 5-HT3 receptor antagonists such as ondansetron can interact with OCTs and MATEs. Initially, the inhibitory potencies of ondansetron on metformin accumulation mediated by OCT2 and MATEs were determined in stable HEK293 cells expressing these transporters [71], showing that ondansetron is a potent MATE inhibitor and a mild OCT2 inhibitor. Furthermore, *in vivo* experiments showed that cisplatin caused much more severe nephrotoxicity in Mate1-/- mice in comparison to wild-type mice [71]. Similarly, co-treatment with ondansentron and cisplatin in wild-type mice caused increased nephrotoxicity as evidenced by increased levels molecular biomarkers of kidney injury and by more severe pathohistological changes in kidney tissues [71].

Overall, these studies demonstrate that in humans the interplay between OCT2 and MATE may affect the net renal secretion of shared drug substrates, including Pt drugs, but further investigation will be necessary to elucidate fully the complex pathways of interaction.

### 2.2. Experimental Anticancer Metal Compounds

#### 2.2.1. Ruthenium Complexes

Concerning the most studied coordination Ru(III) compounds, the previously mentioned KP1019 and NKP-1339 are administered intravenously and, therefore, their interactions with serum proteins are of great relevance. In fact, several studies have shown strong affinities for both compounds to proteins in the bloodstream, particularly serum albumin and transferring [3]. Accordingly, it has been suggested that these proteins act not only as ruthenium carriers and delivery systems, but are also essential for tumor targeting. While binding to albumin may contribute to the targeted delivery of ruthenium compounds to cancer tissues due to the phenomenon known as Enhanced Permeability and Retention (EPR) effect [72] (due to the combination of leaky blood capillaries and lack of lymphatic drainage in tumors), binding to transferrin may constitute an “active” targeting route. In fact, the iron transport protein transferrin (Tf) is crucial in tumor development since highly proliferative tumors have higher demand for iron than normal tissues, resulting in the overexpression of the transferrin receptor (CD71).

Numerous studies [73] describing the reactivity of KP1019 and NKP-1339 with Tf, as well as the accumulation of the compounds in cancer cells, support the hypothesis that selective delivery of these Ru(III) compounds occurs into the malignant tissue via Tf followed by cellular uptake via Tf receptors. The receptor-mediated incorporation of Tf results in the formation of endosomes having low pH (*ca.* 5.5) with respect to the physiological one, which is supposed to trigger the release of the ruthenium compounds inside the cells [74]. Unfortunately, to the best of our knowledge, no actual validation of such uptake pathway has been reported so far for ruthenium compounds.

In 2005 Heffeter, Keppler *et al*., investigated whether the ABC family of drug transporters may lead to resistance against KP1019-induced cytotoxicity *in vitro* [3,75]. Thus, KP1019 was tested against a panel of chemosensitive cell lines and their chemoresistant sublines expressing defined resistance mechanisms; the results showed that the cytotoxic effects of KP1019 are not substantially hampered by overexpression of the drug resistance proteins multidrug resistance-related protein 1 (MDR1), breast cancer resistance protein (BCRP), and lung resistance protein (LRP) or the transferrin receptor, and only marginally by the cellular p53 status. In contrast, P-glycoprotein overexpression reduced KP1019 activity weakly but significantly (up to 2-fold). P-glycoprotein (P-gp)-related resistance was based on reduced intracellular KP1019 accumulation and was reversible by known P-glycoprotein modulators.

To analyze whether KP1019 directly interacts with P-gp, the impact on the P-glycoprotein ATPase activity was measured in the presence of ouabain, EGTA, and sodium azide to block the membrane-bound Na^+^/K^+^, Ca^2+^, and mitochondrial ATPases [75]. Interestingly, KP1019 dose-dependently inhibited ATPase activity of P-gp. Furthermore, it potently blocked P-gp-mediated rhodamine 123 efflux under serum-free conditions (EC_50_, 8 μM), however, with reduced activity at increased serum concentrations (EC_50_ at 10% serum, 35 μM).

Many P-gp substrates also act as P-gp modulators and competitively inhibit the efflux of other substrate drugs [76]. To test whether KP1019 was able to modulate P-gp-mediated resistance, the compound was administered to P-gp-overexpressing cells together with the two well characterized P-gp substrates daunomycin and etoposide, as well as cisplatin, which is not transported by P-glycoprotein [75]. Resistance of P-glycoprotein-overexpressing KBC-1 cells against daunomycin but not cisplatin was slightly but significantly reduced when KP1019 was added at low, nontoxic concentrations.

**Figure 3:**
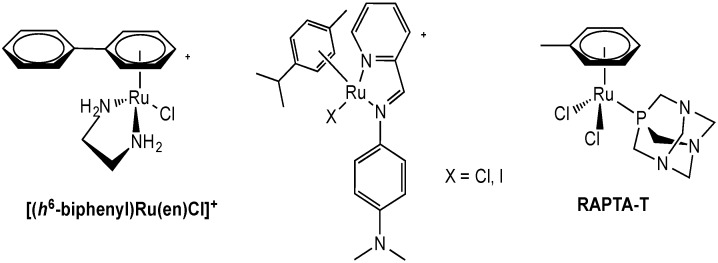
Organometallic Ru(II) arene complexes.

Following the assumption that Ru(II) species may be the active ones following Ru(III) prodrug(s) treatment, Ru(II) complexes were also synthesized and investigated for their anticancer properties. Within this strategic framework, organometallic Ru(II)-arene compounds have been developed, including those containing phosphine, amine, and sulfoxide as co-ligands. These compounds show promising *in vitro* and/or *in vivo* antitumor activities [77,78]. Interestingly, while monofunctional complexes of the type [η^6^-arene)Ru(en)X]^+^ (en = ethylenediamine or derivatives, X = halide) (Figure 3) exhibit high cytotoxicity *in vitro* comparable to that of cisplatin, which can be modulated by the arene ligand [79], the bi-functional complexes of the general formula [η^6^-arene)Ru(PTA)X_2_] (PTA = 1,3,5-triaza-7-phosphaadamantane, X = halide) (RAPTA, Figure 3) showed anti-metastatic properties and generally low toxicity as reported for NAMI-A [77]. The pharmacological properties of mono-functional Ru(II) complexes have been mainly attributed to their reactivity with nucleic acids leading to DNA damage and cell death, even if a different DNA mode of binding is observed compared to cisplatin. Instead, the RAPTA compounds appear to work on molecular targets other than DNA, implying a biochemical mode of action profoundly different from that of classical Pt anticancer drugs. Indeed, it is likely that the mechanism of action of the RAPTA complexes may involve interactions with critical intracellular or even extracellular proteins [77,80].

Concerning possible transport mechanisms, Sadler *et al*., investigated two iminopyridine ruthenium(II) arene complexes, which differ in their halide ligands, Ru(η^6^-*p*-cymene)(*N*,*N*-dimethyl-*N*'-[(*E*)-pyridine-2-ylmethylidene]benzene-1,4-diamine)X]PF_6_ (X = Cl, I, Figure 3) [81]. Possible pathways for the accumulation of these two organometallics were studied in human ovarian cancer A2780 cells in comparison to cisplatin. Cells were co-incubated with the compounds and different concentrations of one of the following substances: (a) verapamil (competitor for efflux via P-gp); (b) oubain (inhibition of Na^+^/K^+^ pump); (c) CuCl_2_ (competitor for transport via hCtr1); (d) antimycin A (ATP depletion); (e) amphotericin B (membrane disruption and model for protein-mediated transport) and f) methyl ß-cyclodextrin (caveolae endocytosis pathway); the amount of metal (Ru/Pt) uptake was determined by ICP-MS. Interestingly, by changing from a chloro to an iodo ligand, the mechanism of uptake varied from being active to mainly passive, respectively. Nevertheless, competition experiments with Cu^2+^ (200 μM) indicate that, while Pt accumulation from cisplatin treatment is reduced by *ca.* 40%, accumulation of Ru is only reduced by 26% for the chloro complex [81]. Instead, Ru uptake for the iodo derivative is reduced to a third of its original value. The experiments using the cardiac glycoside oubain suggest that the membrane potential of the cell and the corresponding electrochemical gradient are key determinants of Ru compound uptake. The role of protein-mediated transport in the cellular accumulation of Pt and Ru drugs was also investigated, co-incubating cells with variable concentrations of amphotericin B, which forms pores in the cellular membrane. These pores, permeable to water and non-electrolytes, may give rise to increased drug influx and therefore higher cellular accumulation. In this case, the obtained results showed no effect on the uptake of the chloro derivative, but had a marked influence on the iodo analogue, in accordance with the results of the temperature-dependent uptake studies, according to which passive diffusion of this complex through the cell membrane is involved. Finally, endocytosis pathways appear not to be involved in the uptake of Ru complexes as shown by co-incubation experiments with methyl β-cyclodextrin [81].

Concerning efflux mechanisms, using verapamil, it was possible to impair the efflux of both ruthenium complexes most likely acting on a P-gp dependent efflux pathway [81]. In fact, verapamil, an l-type calcium channel blocker, effectively abrogates P-gp mediated active efflux of anticancer drugs in ovarian cancer cells by competitive inhibition of drug transport, and is capable of reversing multi-drug resistance [81]. Co-treatment with antimycin A, which can deplete ATP levels and therefore affects the ATP-dependent efflux pump, increased the accumulation of the chloro complex (consistent with verapamil results), but not that of the iodo derivative [81].

Overall, these results evidence a highly complex network of accumulation pathways for Ru compounds which are dependent on various determinants including the cell type investigated, the type of ligand set stabilizing the metal center, the oxidation state of the metal, the possible effects of the compounds on the transporters distribution and expression, as well as the metal complex speciation pathways. For example, reactions with glutathione may lead to thiolate as well as sulfenate and sulfinate derivatives, which may be recognized by different efflux transporters, as in the case of products from reaction of cisplatin with GSH.

#### 2.2.2. Gold Complexes

**Figure 4:**
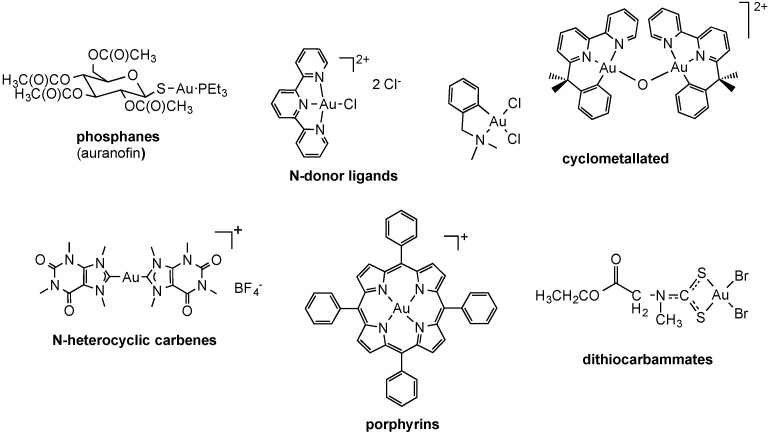
Examples of cytotoxic coordination and organometallic gold(I) and gold(III) compounds.

As mentioned above, gold compounds have attracted increased attention as a source of novel cytotoxic molecules with potential uses in cancer treatment [5,82,83]. Indeed, both gold(I) and gold(III) complexes have been widely investigated and were found to induce important anticancer effects *in vitro* and *in vivo*. Among the various families of gold-based coordination compounds the following were extensively studied for their biological effects: as well as gold(III) complexes with N-donor ligands. Representative structures for each family are presented in Figure 4.

It is worth mentioning that, in spite of their promising anticancer effects, the risk in developing gold compounds for biological applications is that they may be characterized by a remarkable oxidizing character (particularly Au(III)), especially within the fairly reducing intracellular milieu. Therefore, in order to guarantee more controlled chemical speciation in an aqueous environment, different types of organometallic gold complexes have been synthesized in which the presence of a direct carbon**-**gold bond greatly stabilizes the gold(I)/(III) redox couple [84]. Thus, a variety of cyclometallated gold(III) complexes of nitrogen donor ligands have been synthesized, featuring both bidentate C,N- and terdentate C,N,N-, C,N,C- and N,C,N-donor ligands, with either five- or six-membered C,N rings (Figure 4) [84]. Similarly, organometallic gold(I)/gold(III) complexes with *N*-heterocyclic carbene (NHC) ligands have also been explored as cytotoxic agents (Figure 4) [84,85]. In general, both organometallic gold(I) and gold(III) centers have increased stability with respect to classical gold-based coordination complexes and are extremely suitable to design gold compounds still acting as pro-drugs, but in which the redox properties and ligand exchange reactions can be modulated to achieve selective activation in diseased cells.

**Figure 5:**
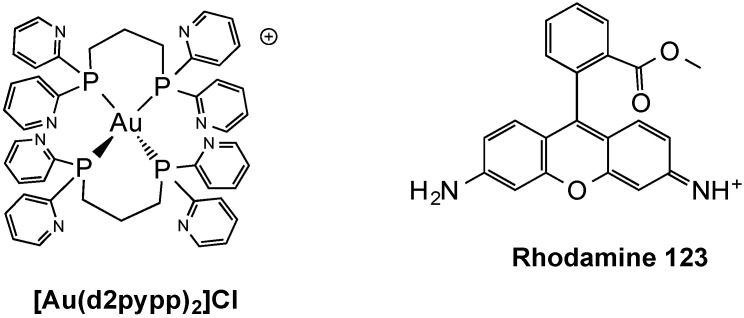
Examples of delocalized lipophilic cations (DLCs) including a Au(I) complex.

Concerning Au(I) phosphine compounds, which have shown early promise as anticancer drugs, they can be divided into two distinct classes based on coordination chemistry and propensity to undergo ligand exchange reactions with biological thiols and selenols. These are (i) neutral, linear, two-coordinate complexes such as auranofin; and (ii) lipophilic, cationic, bis-chelated tetrahedrally four-coordinate Au(I) diphosphine complexes such as [Au(dppe)_2_]^+^ (dppe = 1,2-bis(diphenylphosphino)ethane). Although evidence suggests that the mechanism of antitumor activity of the two classes is different, mitochondria have been implicated as targets in both cases [86]. Notably, among the members of the latter family, a bis-chelated Au(I) complex of the water-soluble bidentate pyridylphosphine ligand 1,3-bis(di-2-pyridylphosphino)propane (d2pypp), namely, [Au(d2pypp)_2_]Cl (Figure 5) [87], was designed with a lipophilicity (log *p* = −0.46) in the optimal range derived from predictive models for the selective accumulation of the so-called DLCs (Delocalized Lipophilic Cations) in cancer cells based on lipophilicity [88].

It is worth mentioning that DLCs have been explored as an approach to cancer chemotherapy that exploits their selective accumulation in mitochondria of cancer cells as a consequence of the elevated transmembrane mitochondrial potential ∆ψ_m_ [89]. In fact, DLCs can pass easily through the lipid bilayer and their positive charge then directs them to the mitochondria where they accumulate at significantly higher concentrations than in the cytoplasm, owing to the large ∆ψ_m_ generated by the respiratory chain [90]. While DLCs share a common mechanism for mitochondrial accumulation, their structures are diverse and consequently their mechanism of antitumor action and mitochondrial targets may vary.

Notably, in addition to the increased Δψ_m_, some cancer cells have been found to have higher plasma membrane potentials (Δψ_p_), which further contributes to the increased uptake of DLCs by cancer cells [91]. Since all cells generate an electrical gradient, which is negative on the inside of the cell, Δψ_p_ could act as an attractive force for intracellular accumulation of agents of this nature. Notably, preferential accumulation and toxicity of DLCs has been demonstrated in a number of different carcinoma cell lines as compared to normal epithelial cell types. As an example, Chen and coworkers studied more than 200 epithelial-derived cell lines and found that carcinoma cells consistently had a higher level of uptake and retention of Rhodamine-123 (Rh-123) than normal human epithelial cells [92]. Taken together, to date there is direct evidence supporting a link between the retention of positively charged compounds in tumor cells and increased Δψ_p_ [93], while the role of Δψ_m_ in this phenomenon remains to be further investigated [94]. Nevertheless, recently Ott and coworkers evaluated the cellular uptake as well as the biodistribution of a series of Au(I) NHC complexes by atomic absorption spectroscopy [95]. According to their results, a marked mitochondrial accumulation of compound triphenylphosphine-[1,3-diethylbenzimidazol-2-ylidene]gold(I) could be related to its high cellular uptake and higher lipophilic cationic character within the tested derivatives [95].

Apart from the specific case of DLC-type Au(I) complexes, the mechanisms of uptake and accumulation of gold compounds in cells are, in general, poorly established. Since the interaction with thiols is an important parameter in the biochemistry of gold-based drugs, a “thiol shuttle” model involving binding of gold compounds to the surface exposed Cys-34 of serum albumin has been proposed. According to this model, cellular association, intracellular distribution, and efflux of gold complexes via sequential thiol exchange reactions, also involving reversible binding to serum albumin, may take place [96].

**Figure 6:**
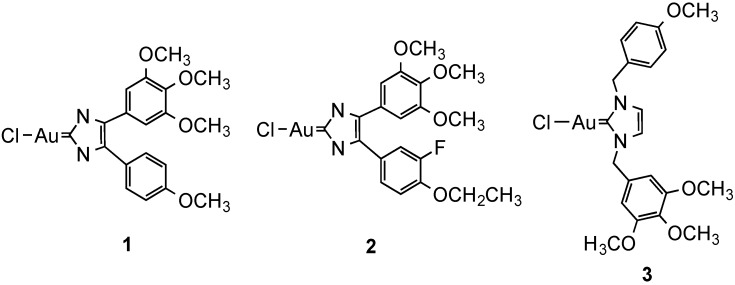
Au(I) NHC complexes chloride-[1,3-dimethyl-4,5-diarylimidazol-2-ylidene]gold(I) **1**–**2** and chloride-[1,3-dibenzylimidazol-2-ylidene]gold(I) **3**.

Recently, neutral heterocyclic Au(I) NHC complexes of the type chloro-[1,3-dimethyl-4,5-diarylimidazol-2-ylidene]gold(I) **1**–**2** and chloro-[1,3-dibenzylimidazol-2-ylidene]gold(I) **3** reported in Figure 6, modeled on the vascular disrupting anticancer drug combrestatin A-4, were studied for their biological properties in cancer cells [97]. The compounds were cytotoxic in the μM range and with distinct selectivity for certain cell lines. The contribution of the various routes of uptake of the test compounds **1**–**3** was assessed in 518A2 melanoma cells by pre-treating them with non-toxic concentrations of specific inhibitors or competitors of the individual uptake processes. Thus, cimetidine (inhibitor) and TEA (competitor) were used to assess possible transport via hOCT1-2, while CuCl_2_ (competitor) was employed to investigate hCtr1 related transport. Adding oubaine as inhibitor of the Na^+^/K^+^ pump could slow down endocytotic processes dependent on a sodium gradient. Subsequently, the cytotoxic effects were measured via MTT assays after 3 h incubation with the compounds, following 2 h pre-incubation with inhibitor/competitor. The results suggest that cellular uptake for all tested gold complexes occurs mainly via the OCT transporters, and for complex **2** also via hCtr1. In addition, complexes **2** and **3** were also internalized via the Na^+^/K^+^-dependent endocytosis. Interestingly, complex **1** did not show particular specificity for any of the investigated transport pathways. Unfortunately, these results were not complemented by measurements of the gold uptake in cell extracts, which may have ruled out other effects induced by the compounds’ treatment on the transporters expression and distribution.

#### 2.2.3. Iridium Complexes

**Figure 7:**
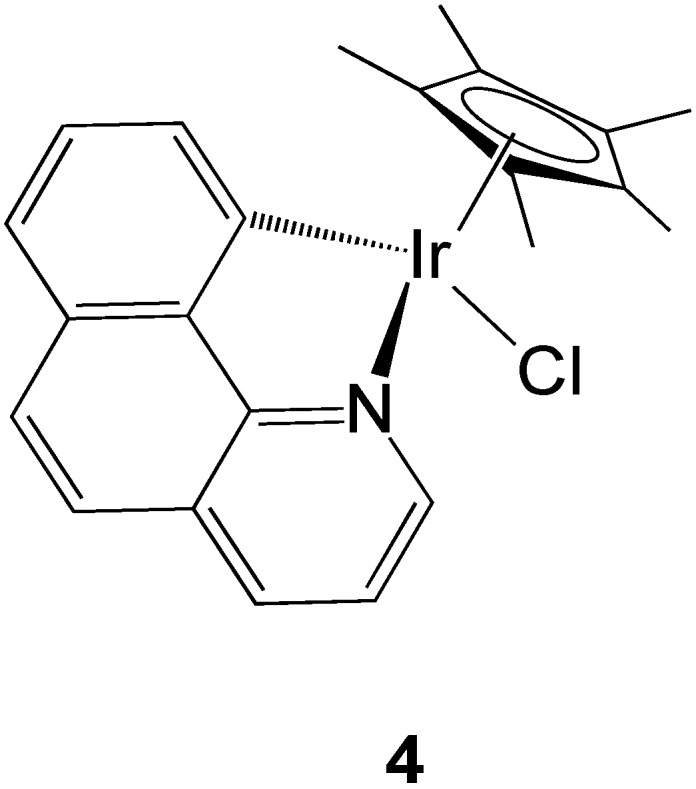
Schematic drawing of the organometallic compound [(*ƞ*^5^-Cp*)(Ir)(7,8-benzoquinoline)Cl].

Iridium compounds are another promising class of experimental metal-based cytotoxic agents [98]. In a recently published paper from Novohradsky *et al*., the mechanisms of accumulation of new cytotoxic organometallic iridium(III) complexes were investigated in cancer cells [99]. Specifically, a half-sandwich cyclometallated Ir(III) complex [(η^5^-Cp*)(Ir)(7,8-benzoquinoline)Cl] bearing a C^N chelating ligand (Figure 7) was studied for its uptake in ovarian cancer A2780 cells in comparison to cisplatin. Beside temperature dependence experiments, co-incubation with different substrates such as (a) ouabain (facilitated diffusion endocytosis pathway); (b) 2-deoxy-d-glucose and oligomycin (ATP depletion); (c) efflux inhibitors such as verapamil (P-gp inhibitor), reversan (MRP1 inhibitor) and buthionine sulfoximine (inhibition of GSH synthesis); (d) CuCl_2_, as well as e) methyl-β-cyclodextrin (inhibitor of endycytosis pathway) allowed some conclusions.

Overall, passive diffusion seems to be partly involved in the Ir compound uptake, as evinced by the results of the temperature dependence experiments and the blockage of Na^+^/K^+^ ATPases. In fact, co-incubation with ouabain, leads to a decrease in Ir intracellular accumulation. Moreover, the competition experiments with CuCl_2_ suggest the Ctr1 pathway may also be involved in the compound’s uptake, although to a lesser extent. Concerning the possible involvement of efflux systems in the accumulation of the compound, it appeared that the possible efflux transporters P-gp and MRP1 may play a role, as may formation of GSH-Ir conjugates. Not surprisingly, verapamil and reversan do not restore cisplatin sensitivity since the drug is not recognized by either P-gp [100] or MRP1 [101]. Moreover, it could be observed that the endocytotic pathway does not play a role in Ir accumulation.

#### 2.2.4. Transporter-Targeted Anticancer Metal Compounds

Several studies report on the possibility for metal compounds to be derivatized with biomolecules for different applications [102]. Thus, as a strategy to enhance compound uptake via specific peptide transporters, anchoring of metal centers to peptides has been attempted; Fregona *et al*., reported on gold(III) dithiocarbammate complexes with peptide-based ligands to achieve carrier-mediated delivery of the compounds in cancer cells via peptide transporters (PEPT) [103]. Two peptide transporters, PEPT1 and PEPT2, have been identified in mammals, which are present predominantly in epithelial cells of the small intestine, bile duct, mammary glands, lung, choroid plexus, and kidney but are also localized in other tissues (pancreas, liver, gastrointestinal tract) and, intriguingly, appear to be overexpressed in certain types of tumors. A unique feature of such transporters is their capability for sequence independent transport of most possible di- and tripeptides inside the cells. Moreover, they are stereoselective toward peptides containing l-enantiomers of amino acids.

**Figure 8:**
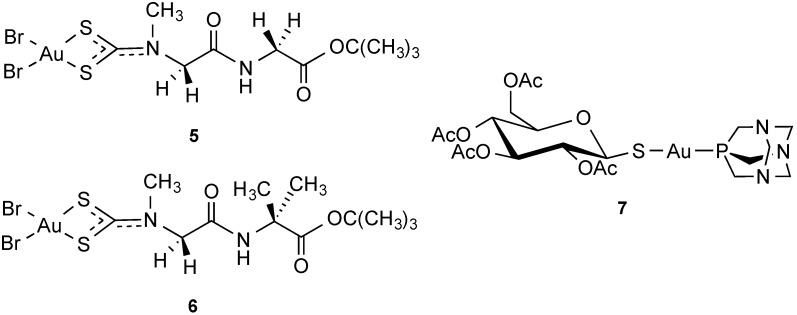
Au(III) dipeptidedithiocarbamato and Au(I)-thiosugar complexes.

Thus, compounds of the type [Au(III)Cl_2_(dpdtc)] (dpdtc = dipeptidedithiocarbamate, **5**–**6**) reported in Figure 8 were designed which could both preserve the antitumor properties and reduce toxic and nephrotoxic side-effects of the previously reported gold(III) analogues lacking the peptide moiety, together with an enhanced bioavailability and tumor selectivity due to the dipeptide-mediated cellular internalization provided by PEPTs [104]. The compounds showed promising antiproliferative effects in a panel of human cancer cells (PC3, DU145, 2008, C13, and L540), reporting IC_50_ values much lower than those of cisplatin. Remarkably, the gold compounds also showed no cross-resistance with cisplatin itself and proved to inhibit tumor cell proliferation by inducing either apoptosis or late apoptosis/necrosis, depending on the cell lines. More recently, the same compounds were proven effective in inhibiting tumor growth in breast cancer xenograft models, and proved to be potent inhibitors of the proteasome [105]. Unfortunately, the mechanisms of uptake of the compounds via PEPT was not investigated and further studies are necessary to validate the proposed strategy for enhanced gold(III) peptidomimetics transport in tumors.

Interestingly, in recent years, several examples of carbohydrate compounds have been developed for diverse medicinal applications, ranging from compounds with antibiotic, antiviral, or fungicidal activity and anticancer compounds [106]. Within this frame, Au(I) complexes with thiosugar ligands, analogues of auranofin (Figure 8, compound **7**), were shown to have an increased uptake in cancer cells [107]. It is possible that the 1-thio-β-d-glucose-2,3,4,6-tetraacetate ligand in the compound is acting as a true substrate for the glucose active-transport system (via GLUT1 transporters), enhancing the uptake of the metal compound itself.

## 3. Conclusions and Perspectives

Pt-based anti-cancer drugs are effective pharmaceuticals and are still among the most widely used agents against malignancies. In parallel to the preparation and screening of new metal complexes as potential anticancer agents, extensive efforts must be directed toward elucidating their mechanism of accumulation in cancerous and non-tumorigenic tissues. Most importantly, a systematic investigational approach should be developed in order to characterize metal compound uptake, efflux and biodistribution in cells. Knowledge of the involvement of metallodrugs transporters could be exploited to develop appropriate intervention schedules. Although *in vitro* models, as those applied in the studies mentioned above, appear to be more suitable and easy to handle for a first approach to the problem, they are characterized by several limitations, including the absence of an extracellular matrix component and of 3D cell-cell interactions, high variability of results depending on the protocols of culture used in each lab, biased results toward cytotoxic agents and poor correlation with clinical efficacy. Therefore, new pre-clinical methodologies should be explored making use of tissues samples, such as the so-called Precision Cut Tissue Slices (PCTS) technique [108], in which viable slices from explants, from healthy or cancerous tissues, can be considered as mini-models of organs in which cells of different types are present in their natural environment, especially concerning cell-cell and cell-matrix interactions.

Overall, future developments in metal-containing agents and chemotherapeutic regimens should focus on novel delivery mechanisms with emphasis on improving the uptake and proper distribution of the existing metallodrugs by e.g., liposomal formulation of cisplatin-like drugs or by binding the Pt compounds to physiological (carrier) proteins to enhance their uptake.
